# Lipid Droplets from Plants and Microalgae: Characteristics, Extractions, and Applications

**DOI:** 10.3390/biology12040594

**Published:** 2023-04-13

**Authors:** Kaiwei Xu, Wen Zou, Biao Peng, Chao Guo, Xiaotong Zou

**Affiliations:** 1 Institute of Systems Security and Control, College of Computer Science and Technology, Xi’an University of Science and Technology, Xi’an 710054, China; xukaiwei12@163.com; 2 Shaanxi Provincial Key Laboratory of Land Consolidation, Chang’an University, Xi’an 710074, China; 3 State Owned SIDA Machinery Manufacturing, Xianyang 712201, China; 4 Key Laboratory of Degraded and Unused Land Consolidation Engineering, Ministry of Natural Resources, Xi’an 710021, China; 5 Faculty of Printing, Packaging Engineering and Digital Media Technology, Xi’an University of Technology, Xi’an 710048, China

**Keywords:** lipid droplets, plants, microalgae, extraction strategy, triacylglycerols

## Abstract

**Simple Summary:**

Lipid droplets (LDs) are cellular organelles that are involved in diverse biological processes. They are composed of a neutral lipid core surrounded by a phospholipid monolayer membrane. LDs are found in many different types of cells and organisms, and play important roles in energy metabolism, signaling, and lipid storage. Recent research has led to a better understanding of the formation, turnover, and functions of LDs, as well as their potential applications in fields such as biofuels, food additives, and cosmetics. However, there are still many questions to be answered in order to fully exploit the potential of LDs, such as understanding the metabolic differences between species and strains, and uncovering the precise functions and relationships of LDs and their associated proteins. Moreover, extracting LDs from plants and microalgae for various applications remains a challenge, with different methods having different advantages and limitations, depending on the intended use. Consequently, further research into LDs could lead to innovative and sustainable solutions for a variety of industries and applications.

**Abstract:**

Plant and algal LDs are gaining popularity as a promising non-chemical technology for the production of lipids and oils. In general, these organelles are composed of a neutral lipid core surrounded by a phospholipid monolayer and various surface-associated proteins. Many studies have shown that LDs are involved in numerous biological processes such as lipid trafficking and signaling, membrane remodeling, and intercellular organelle communications. To fully exploit the potential of LDs for scientific research and commercial applications, it is important to develop suitable extraction processes that preserve their properties and functions. However, research on LD extraction strategies is limited. This review first describes recent progress in understanding the characteristics of LDs, and then systematically introduces LD extraction strategies. Finally, the potential functions and applications of LDs in various fields are discussed. Overall, this review provides valuable insights into the properties and functions of LDs, as well as potential approaches for their extraction and utilization. It is hoped that these findings will inspire further research and innovation in the field of LD-based technology.

## 1. Introduction

Lipid droplets (LDs), also known as oil bodies, lipid bodies, lipid particles, oil globules, cytoplasmic inclusions, spherosomes, or oleosomes, are the major storage organelles of lipids, such as neutral lipids, triacylglycerols, and smaller amounts of polar lipids in both eukaryotic and prokaryotic cells [1,2]. Lipids play important roles in cellular processes which include but are not limited to membrane remodeling, inter-organelle communications, and energy homeostasis [3,4,5]. Originating from the endoplasmic reticulum (ER), LDs are recognized as dynamic organelles with unique lipid and protein compositions, sizes, and morphology, and they respond to stress conditions and are involved in interactions with other cell organelles [1,6,7].

LDs can be isolated from oil-rich tissues and organs in terrestrial plants, including seeds, pollen, the spores of mosses, roots, and leaves [8,9,10,11]. LDs are also widely represented in microalgal and protist species [12,13,14]. The basic physiological function of LDs in seeds is as a store of carbon and energy. During the seeds’ development, the large stores of lipids accumulated in the embryonic tissue are packaged into LDs. During germination, LDs can provide the necessary carbon source and energy for the seeds. Additionally, it has been reported that LDs may also be involved in other physiological processes in seeds, such as preventing germination under favorable conditions for a period of time after the seeds have dried [1]. Similar to seeds, pollen grains accumulate LDs during maturation, which can provide a source of carbon and energy that drive the germination of pollen and the growth of pollen tubes, and the LDs in pollen and pollen tubes play a key role in the fertilization process of flowering plants [15]. The content of LDs in leaves is generally much lower than that in seeds and pollen, but can easily change with a change in the external conditions [16]. Similar to leaves, various microalgae have LDs in their cells, and their content can be changed under stress conditions. LDs are also involved in many growth and development processes of microalgae [1].

LDs have been exploited for numerous applications in biotechnology as feedstocks for the production of biofuels, vaccine formulations, carriers of drug delivery system, food supplements, cosmetics, and personal care products [17,18,19]. To promote the wide use of LDs at a large scale, it is necessary to establish an easy extraction technology. Hence, efforts towards the isolation of LD have been made. This review provides information about LDs in plants and microalgae with a focus on their properties, function, extraction strategies, and the factors affecting the stability of LDs, as well as the potential applications of LDs.

## 2. Characteristics of Lipid Droplets

**Figure 1:**
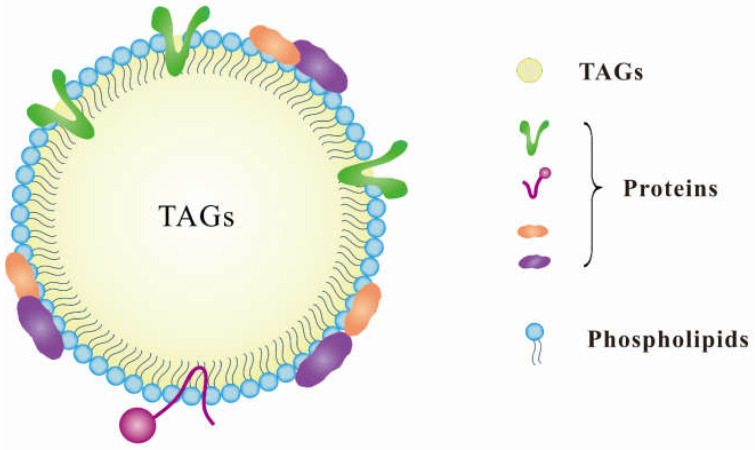
Schematic model of an LD’s structure. Abbreviations: TAGs, triacylglycerols.

Compared with other organelles, LDs have a relatively simple structure containing a hydrophobic core, mainly comprising triacylglycerols (TAGs) and surrounded by a phospholipid monolayer and various surface-associated proteins (Figure 1). In different organisms, these TAGs are quite different in their fatty acid composition, including the number of carbon atoms, the presence and position of double bonds on the fatty acyl chain, and the position of the glycerol head group. Five common fatty acids are palmitic, stearic, oleic, linoleic, and linolenic acids. LD-associated proteins not only play an extensive role in the formation and degradation processes of LDs, but also play an important role in the interfacial structure of LDs. Alongside proteins, phospholipids also play a role in the unique structure and stability of the interface due to the dense crystalline (hard) phase. This LD model was first characterized in mature maize seeds [20], followed by their characterization in diverse organisms [1,4,21]. In addition to TAGs, the LD core can contain sterols, wax esters, steryl esters, or carotenoids in some organisms [14,22,23,24]. For examples, phytosterol and fatty acid esters accounted for 0.3% of Arabidopsis seed oil and 24% of neutral lipid in tobacco pollen [1]. The accumulation of squalene and carotenoids by thraustochytrids accounted for 13 mg/g and 72 µg/g of the cells’ dry weight [14]. In general, plant and microalgal LDs contain about 80–98% (*w*/*w*) TAGs, 1–10% phospholipids, and 1–4% proteins [2,24]. The thickness of the LD membrane composed of the phospholipids monolayer is about 0.9 nm, which contributes 1–2% of the cells’ total lipid content. Phospholipids mainly contain phosphatidylcholine, phosphatidylserine, phosphatidylethanolamine, and phosphatidylinositol [1,25,26,27] (Table 1).

**Table 1:** The composition of lipid droplets extracted from various species.

Organisms		TAGs	Protein	Phospholipid	Others (Sterols, Wax Esters, Steryl Esters, Carotenoids)	References
Plants	Soybean	<40.1%	8.8%	Na*	Na*	[28]
	Peanuts	<98.1%	1.27%	0.77%	Na*	[29]
	Sunflower seed	<92.6%	7.3%	Na*	Na*	[30]
	Maize	97.66%	1.43%	0.91%	Na*	[20]
	Rapeseed	94.21%	3.46%	1.97%	0.36%	[27]
	Coconut	<38.2%	4.1%	Na*	0.15%	[31]
	Safflower	97%	2.5%	0.7%	Na*	[32]
	Cotton	96.99%	1.70%	1.18%	0.13%	[27]
	Flax	97.65%	1.34%	0.90%	0.11%	[27]
	Sesame	97.37%	0.59%	0.57%	0.13%	[27]
	Mustard	94.64%	3.25%	1.60%	0.17%	[27]
Algae	*Chlamydomonas reinhardtii*	85–95%	Na*	<5%	10%	[2]
	*Dunaliella salina*	>90%	Na*	<10%	Na*	[33]
	*Thraustochytrid*	81%	0.5%	Na*	Na*	[14]
	*Diatom*	58%	2.3%	Na*	Na*	[34]

Na*: not available.

**Figure 2:**
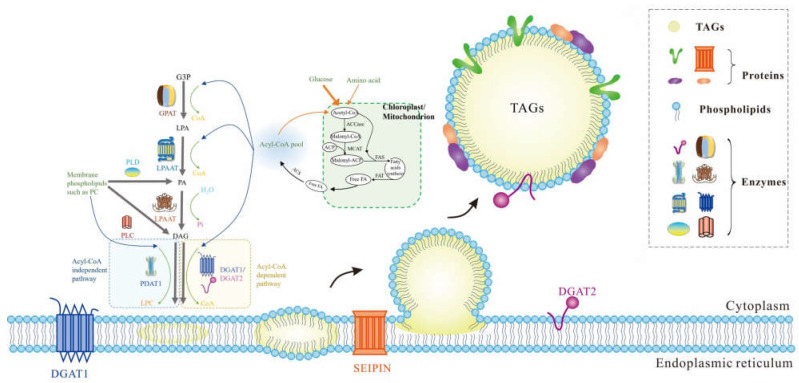
Schematic model of the synthesis of TAG and the formation of LDs. Abbreviations: LD, lipid droplet; TAG, triacylglycerol; ACCase, acetyl-CoA carboxylase; ACP, acyl carrier protein; MCAT, malonyl-CoA: ACP transacylase; FAS, fatty acid synthase complex; FAT, fatty acyl-ACP thioesterases; G3P, glycerol-3-phosphate; GPAT, glycerol-3-phosphate acyltransferase; LPA, lysophosphatidic acid; LPAAT, lysophosphatidic acid acyltransferase; PA, phosphatidic acid; PAP, phosphatidic acid phosphatase; DAG, diacylglycerol; DGAT, diacylglycerol acyltransferase; PDAT, phospholipid: diacylglycerol acyltransferase; PC, phosphatidylcholine; PLC, phospholipase C; PLC, phospholipase D. The figure was adapted from [4].

Currently, most of what we know about lipid biosynthesis pathways in vivo comes from studies on yeast and *Arabidopsis thaliana* [35,36,37,38,39]. The lipid biosynthesis pathway of microalgae is very similar to that of higher plants [40,41]. The main synthesis pathways are as follows (Figure 2).

Acetyl-CoA is a major starting material and substrate for the synthesis of fatty acids, and can be produced by fatty acid oxidation, glycolysis, and degradation of amino acids [42,43]. Among these processes, the acetyl-CoA in cells during the growth phase is thought to be largely derived from the glycolysis pathway of pyruvate [42,43,44]. In this pathway, glucose is broken down into pyruvate in the cytoplasmic matrix through the catalysis of enzymes, and then pyruvate is converted to acetyl-CoA by the pyruvate dehydrogenase complex (PDHC) in mitochondria and chloroplasts. Acetyl-CoA is catalyzed by acetyl-CoA carboxylase (ACCase) to form malonyl-CoA, then malonyl-CoA is catalyzed by malonyl-CoA: ACP transacylase (MCAT) to form malonyl-ACP. After that, through catalysis of the fatty acid synthase complex (FAS), malonyl-ACP and acetyl-CoA are used as substrates for continuous polymerization, adding two carbon chains in each cycle. Thus, fatty acid-ACPs with different carbon chain lengths are formed. During this process, saturated fatty acid-ACP can be further catalyzed by fatty acid desaturase to form unsaturated fatty acid-ACP. Finally, fatty acid-ACP is released from acyl carrier proteins under the action of fatty acyl-ACP thioesterases (FAT), and free fatty acid is formed [42,45,46,47]. Free fatty acid can be catalyzed by acyl-CoA synthase (ACS) to form acyl-CoA and then transported to the endoplasmic reticulum for further processing to form TAG.

In the endoplasmic reticulum, the synthesis of TAGs in land plant species include pathways that are dependent on or independent of acyl-CoA [1,2,48] (Figure 2). In general, the biosynthesis of TAG starts with the esterification of glycerol-3-phosphate (G3P) and acyl-CoA is catalyzed by glycerol-3-phosphate acyltransferase (GPAT), producing lysophosphatidic acid (LPA). Then LPA and acyl-CoA are converted to phosphatidic acid (PA) through the catalysis of lysophosphatidic acid acyltransferase (LPAAT). In the penultimate step, PA is dephosphorylated by phosphatidic acid phosphatase (PAP) to generate diacylglycerol (DAG). In the acyl-CoA-dependent pathway, the final step is catalyzed by diacylglycerol acyltransferases (DGATs) (i.e., DGAT1 and DGAT 2), which convert DAG and acyl-CoA into TAGs. In the acyl-CoA-independent pathway, the final step is catalyzed by phospholipid: diacylglycerol acyltransferase (PDAT), which uses phosphatidylcholine (PC) as an acyl donor for the formation of TAG from DAG. Additionally, DAG can also be synthesized from membrane phospholipids by phospholipase C (PLC) or phospholipase D (PLD) and PAP. It is noted that many organisms have distinct classes of DGATs that have no or very little homology to each other but catalyze the same biochemical reaction [49]. DGAT1 is localized to the endoplasmic reticulum (ER), while DGAT2 is inserted into leaflet of the ER membrane and can diffuse onto the surface of LDs [50]. One important non-enzymatic player in the formation of LD is the SEIPIN localized at the ER–LD junction sites, where they adjust the appropriate size and formation of LDs. They also help to stabilize ER–LD connections [1].

In plants and microalgae, LDs range from 0.2 to 2.5 µm in diameter, with an equilibrium density of less than 1.09 g cm^−3^ [11,24,51]. In plants, the average sizes and their zeta potentials can vary in different cells, and these respond to developmental and environmental conditions as well as stress [2,24]. Additionally, the zeta potential of LDs in plants and microalgae can also vary depending on the species, pH, and/or ionic strength. For example, the zeta potential of LDs from thraustochytrids changed from −51 mV at pH 8 to +4 mV at pH 2.5 [14,52]. The zeta potential of LDs freshly isolated from soybean averaged −14.4 mV at pH 7 to +31.7 mV at pH 2 [53]. Generally, LDs have an isoelectric point less than pH 7 because the proteins wrap around the LDs. The information on LDs from plants and microalgae are summarized in Table 2.

## 3. Features of Proteins Associated with Lipid Droplets

LDs are an important source of cellular energy that is degraded during germination, senescence, and starvation by lipases, which hydrolyzes TAGs into fatty acids. Research has shown that LD-associated proteins contain a binding site for lipases [51]. Zienkiewicz et al. [63] found a striking difference between the dynamic nature of LDs from plants and algae. The LDs in plants are irreversibly mobilized during the germination of seeds or pollen, while microalgae use LDs as transient reservoirs that are quickly degraded in response to environmental stresses such as N deprivation, and are restored by N resupply.

LD proteins can be grouped according to their functions, structure, enzymes, membrane protein trafficking, and signaling [51]. These LD proteins are involved in the formation, maintenance, and/or turnover of LDs. There are two types of proteins (oleosin and algal surface LD proteins) which are long enough to insert into TAGs [51,64]. The other proteins, due to the lack of a short or non-hydrophobic segment, wrap around the LD membrane. Table 3 lists the characteristics of LD-associated proteins in plants and microalgae. The functions of these proteins have been described in detail in previous research [33,51,65,66].

The field of LD cell biology hypothesizes that cells use specific protein machinery to control biophysical processes, making it crucial to identify these proteins to elucidate their functions and means of regulation. Currently, several identification methods exist, including the following. First, HECTAR, which is based on a statistical sequence-based predictive algorithm, can be used to identify signal peptides and Type II signal anchor sequences in proteins from eukaryotic organisms [67]. Secondly, both green fluorescent protein (GFP) and immunogold labelling can locate LD-associated proteins through visualization, with the latter being more subtle. Third, isolated proteins that can be identified by liquid chromatography coupled with mass spectrometry (LC/MS) or gel electrophoresis, but may be affected by the high lipid content of LD samples and the hydrophobic properties of some proteins. Lastly, suspension trapping (strap) is a new technological method combining the advantages of SDS-based protein extraction, and can also be used for proteomic analyses [68,69]; this method has the potential to become a tool for identifying LD proteins due to its simplicity and robustness.

**Table 3:** Information on LD-associated proteins in plants and microalgae.

Proteins	Function	Molecular Mass	References
Oleosin	Involved in the structure; influence the size and stability of LDs	15–25 kD	[70,71]
Caleosin	Involved in peroxygenase activity	20–30 kD	[72,73]
Steroleosin	Involved in brassinosteroid metabolism	40–41 kD	[74,75]
α-Dioxygenase	Phytoalexin synthesis	~73 kD	[76]
LD-associated protein (LDAP)	Involved in the formation and turnover of LDs; related to stress	~25 kD	[16]
Oil-body-associated protein (OBAP1)	Regulating the size of LDs	~27 kD	[77]
CGI-58	Involved in LDs’ homeostasis	Na*	[78]
PXA1	Involved in LDs’ metabolism and signaling	Na*	[79]
SEIPIN	Modulating the accumulation of TAG and influencing the proliferation of LDs	Na	[80]
Sugar-dependent 1 (SDP1)	Involved in the degradation of LDs	Na*	[81]

Na*: not available.

## 4. Lipid Droplet Extraction Strategies

Conventional methods for extracting LDs from plants and microalgae include physical, chemical, and biological methods, as well as combinations of these techniques.

### 4.1. Physical Method

**Figure 3:**
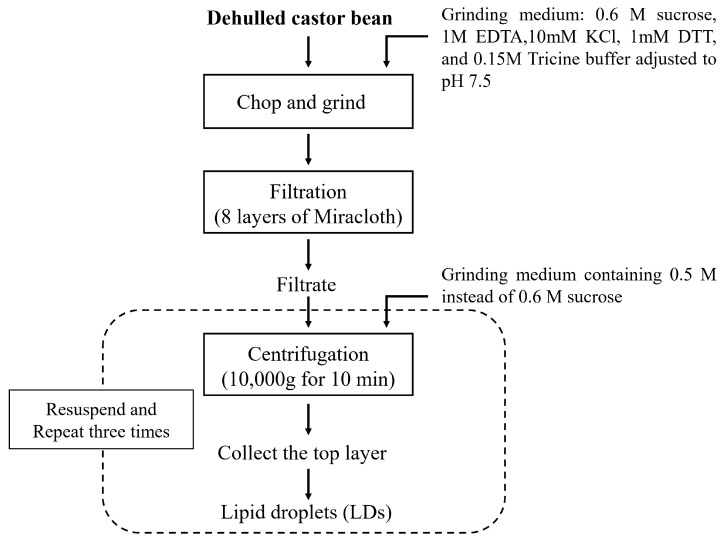
Flow diagram of extracting LDs from castor bean.

LDs can be isolated using a flotation–centrifugation method (see Figure 3). This LD extraction technique was developed for the lipidomic, proteomic, and structural analysis of LDs [20,28]. A flow diagram of this method is shown in Figure 3. To break the cell walls, several mechanical methods can be used, including grinding, pressing, and applying high pressure [20,26,82]. However, during these procedures, LDs can be oxidized in the presence of water and air, and this reaction is catalyzed by lipoxygenase [83]. For this reason, to avoid lipoxygenase, extraction can be performed at low temperatures (such as 4 °C) [20,84].

#### 4.1.1. Grinding

At the laboratory scale, we can use a mortar and pestle to complete the grinding process. This method is also used to extract DNA from biological cells. However, at an industrial scale, this process can use a ball mill system with a rotating cylinder and beads [85]. This system causes direct damage to the cell wall via friction or collision, no matter whether the biomass is wet or dry. Several factors affect the rupturing efficiency of this method, such as the time, pressure, stirring time, and species. The simplicity of the equipment and the operation give advantages to this method. However, this process without repeated grinding and re-extraction may result in less than 45% of the LD yield of full-fat soybean flour due to inadequate rupture of the cellular structure [86]. When the grinding method was used for *Chlorella vulgaris*, a lipid concentration of less than 10% was achieved without the assistance of other methods [87].

#### 4.1.2. Pressing

Mechanical pressing is commonly used to extract seed oils from crops such as soybean, peanut, rapeseed, and sunflower [19,26]. The method can maintain the chemical integrity and/or structure of the substance originally contained in the seeds [88]. Pressing damages the cell walls, releasing the LDs. However, extracting the LDs from microalgae using pressing is not easily achieved, since some algal cells flow on the moisture in microchannels of water, which results in the loss of biomass [89]. The extraction efficiency of this method is significantly dependent on the species selected. For higher plants, the LD yield can reach up to 95 wt% through applying pressing and organic solvent extraction [26], while for microalgae, the extraction efficiency of this method can reach 75% [19]. These results suggest that pressing can be a viable option for bulk oil extraction. However, this method is also time-consuming and requires a large amount of algal biomass.

#### 4.1.3. High-Pressure Homogenization

High-pressure homogenization pumps biomass through a narrow tube at high pressure and then releases the biomass into a low-pressure chamber. The cell disruption process uses high-pressure impingement and a hydraulic shear force [90]. This process has a low risk of thermal degradation and low operational costs. The extraction efficiency of this method depends on the species and the rigidity of the cell walls [91]. It was reported that the cells of *Scenedesmus acutus* were almost completely ruptured [92]. In a study on *Nannochloropsis* sp., this method had the highest efficiency for the extraction of the intracellular components [93]. Although this method is promising, further evaluation and optimization are required at a large scale for the extraction of LDs due to its high energy consumption [19].

Along with the abovementioned methods, the physical methods also include the use of ultrasound, microwave, bead beating, and pulsed electric fields to break the cell walls [94,95,96,97]. The principle of ultrasound is that the ultrasonic waves are transmitted in the liquid medium, which generates alternating low-pressure and high-pressure cycles, where vacuum microbubbles are created and then collapse, which eventually disintegrate the cell membrane/wall [98]. Microwaves use electromagnetic radiation with a frequency from 0.3 to 300 GHz. A microwave uses a non-contact heat source, which interact selectively with polar molecules such as water in the biomass and induces intracellular heating, resulting in damage to the cell membrane and releasing the intracellular substances [99]. The principle behind bead beating is similar to that of grinding and bead mills, which achieve cellular disruption by physically grinding them with the assistance of glass beads [97]. Pulsed electric field is also called electropermeabilization, and it can achieve cell disruption by using short electric pulses under a high electric field [100]. Although these methods have not been used to isolate LDs from plants and microalgae, they have been intensively studied for rupturing algal cells, and should be considered as viable options for extracting LDs from plants and microalgae. The advantages and limitations related to the different physical methods of cell disruption are illustrated in Table 4.

### 4.2. Chemical Methods

Cell disruption by base hydrolysis involves treating cells with a base to extract the intracellular LDs. This method has been studied for isolating LDs from both plants and microalgae over the past few years, and many experiments have proposed the effectiveness of this method for extracting LDs from wet biomass [14,101,102]. Zhao et al. [101] reported that plant seeds (e.g., jicama, sunflower, peanut, castor bean, rapeseed, and sesame) were used for the extraction of LDs at different pH values (6.5–11.0). Many extrinsic proteins contaminated the LDs, with the exception of those of peanut at pH 6.5; they obtained highly purified LDs at pH 11.0. Nham Tran et al. [14] extracted LDs from thraustochytrid cells at pH 12 with different hydraulic retention times. They showed that longer treatment resulted in the accumulation of liquid LDs and solidified LDs, and the highest LD yield reached 34% after a treatment duration of 12 h. Although base hydrolysis has high performance in terms of cell disruption, the use of this method in large-scale processes can increase concerns about the safety and the reactor’s design, since the chemicals are corrosive and react with the target products (LDs). In addition, the neutralization of the base increases the cost, especially for dilute biomass. Therefore, further evaluation of this method should be performed for application at a larger scale.

### 4.3. Biological Methods (Enzymatic Lysis)

Enzymatic cell lysis, an efficient and green method, is widely used in the medical and biological fields, and has been tested for the extraction of LDs from plants and microalgae. The enzymes selectively hydrolyze specific chemical bonds, leading to the rupture of the cell wall. The selection of the enzyme plays an important role affecting cell disruption in this process. In a study on soybeans, four commercial enzymes (Pectinase FE, CX B, GC, and CX 13L) were used to isolate intact LDs, and results showed that the maximum oil extraction yield (60.7%) was achieved by the combination of these enzymes [103]. The effects of individual and combined enzyme treatments on *Nannochloropsis* sp. were also investigated, and a higher lipid yield (73%) was reported for the combined enzymatic treatments [104]. However, the combination of enzymes does not always support cell disruption, since inhibition of the enzymic reaction occurs. For example, compared with the individual enzymatic treatments, a lower lipid yield (12%) was extracted from *Scenedesmus sp.* using a combination of enzymes [105]. Generally, enzymic cell lysis is environmentally friendly and efficient, and it has the potential to replace organic solvents and mechanical methods. However, due to the long reaction time, the strict reaction conditions, and the high costs, large-scale applications of this method need further evaluation.

## 5. Factors Affecting the Stability of Lipid Droplets after Extraction

In general, LDs in the seed have a negative surface charge, which keeps them stable in vivo through electrostatic repulsion. Moreover, several authors have claimed that oleosin proteins coating the LD surface provide the LDs with physical and chemical protection against environmental stresses, such as ionic strength, variations in pH, and fluctuations in temperature [28,106]. However, oleosin is hydrolyzed rapidly during post-germinative growth, with the concomitant conversion of TAGs into fatty acids. In a similar process, during the extraction, processing, storage, transport, and utilization of LDs, lipase, protease, and phospholipase will inevitably induce the degradation of the isolated LDs. This process is also susceptible to changes in the external environment. Therefore, it is crucial to understand their functional performance under different environmental conditions, and then improve their stability for the utilization of LDs in various products.

### 5.1. pH

The pH value is one of the most factors affecting the stability of isolated LDs. In general, the ζ potential of LDs increases from a negative charge to a positive charge when the pH value of the emulsion containing the LDs decreases, with the isoelectric point being around pH 3–6 [14,28,106]. This is because the active protein molecules remain around the LDs after the extraction procedure. For example, the ζ potential of LDs isolated from thraustochytrids changed from −57 mV at pH 8 to +4 mV at pH 2.5, with the isoelectric point being around pH 3 [14]. In addition, when the pH value of this kind of emulsion is well away from the isoelectric point, the mean diameters of the LDs are relatively small, and there is strong electrostatic repulsion between the LDs that prevents them aggregating, which is beneficial for increasing their stability. When the pH is close to the isoelectric point, LD aggregation occurs, forming particles with a large diameter. Regarding the effect of pH on the oxidative stability of LDs, studies have shown that the concentrations of primary and secondary lipid oxidation products (lipid hydroperoxides and TBRAS) increase with a decrease in the pH [53,106,107]. This may be because lipoxygenase plays a crucial role in the oxidation of lipids. This suggests that oxidative stability is greatly improved at a high pH value.

### 5.2. Ionic Strength

Salts are usually added to emulsion beverages to improve their flavor and quality; NaCl is one of them. Previous studies have shown that the ζ potential of LDs decreases with increasing NaCl concentrations, and the mean particle diameters of LDs are relatively small at low NaCl concentrations but increase significantly with increasing NaCl concentrations [56,108]. The explanation for this phenomenon is the ability of a certain concentration of NaCl to create an electrostatic screening effect, leading to a low ζ potential and inducing the aggregation of LDs and the emulsions instability. In addition, divalent cations such as calcium are added to food products as processing aids; they can also affect the stability of LD emulsions. According to the study of White et al. [30], sunflower LD emulsions were relatively stable even at high CaCl_2_ concentrations when the pH value was 3 (<the isoelectric point), but at pH 7, the emulsions were susceptible to CaCl_2_. The particles’ diameter significantly increased at CaCl_2_ concentrations of >5 mM, and the viscosity of the emulsions changed from 0.005 to 0.015 Pa·s when the CaCl_2_ concentrations were increased from 0 to 150 mM. The reason is that the Ca^2+^ ions are the main counter ions at a pH above the isoelectric point, and the divalent ions are more effective at associating with the negatively charged surface of LDs and reducing the electrostatic interactions. Additionally, iron ions are also found in food emulsions, which can lead to acceleration of the decomposition of primary lipid oxidation products, consequently causing concerns about undesirable “off-flavors”. The presence of ferric chloride adversely affected the stability of soybean LDs at a pH of 7 [53]. The effects and mechanism of ferric chloride on the physical and oxidative stability are similar to those of CaCl_2_.

### 5.3. Thermal Treatment

Thermal treatment is commonly used in the processing operations of emulsions, especially in the food industry. Consequently, it is important to explore the effect of thermal treatment on the stability of LDs. Studies have shown that there is no significant change in the particle size of LDs when they are heated above 70 °C. In addition, there was no evidence of aggregation or flocculation in the heated emulsions [28,58]. Meanwhile, there was a change in ζ potential of LDs, probably due to the loss of some extrinsic proteins at the interface; and the magnitude of the change in the ζ potential depends on the heating temperature and the duration of heating [28,55,56,58,107]. In a few words, LDs show good thermal stability. Regarding their oxidative stability, the study of Zhao et al. [107] showed that the content of primary and secondary lipid oxidation products remained stable in all soybean LD emulsions with prolonged storage time after heating rather than increasing without heating. This is because lipoxygenase plays an important role in the exposed LDs to form peroxidation products, and these enzymes are inactivated by heating, resulting in improvements in the oxidative stability of LD emulsions.

## 6. Potential Applications of Lipid Droplets

### 6.1. Potential Food Applications

LD-based natural emulsions have wide applications in the area of dairy foods (yogurt, imitation milk, salad dressings, creams, etc.). For example, LDs isolated from plants and microalgae can replace milk fat globules in yogurt. Compared with cow milk yogurt, the final products contain a similar lipid content (about 3%, *w*/*w*) and are rich in polyunsaturated fatty acids, which further enhance the health benefits [109]. Salad dressings are normally based on egg yolks as an emulsifier. Mixtures with LDs can replace conventional lipids, which can improve the products’ properties such as the flavor, physical stability, and rheological properties [26,110].

### 6.2. Biotechnological Applications

Oleosins are one of the major membrane proteins, consisting of amphipathic N and C termini, and a central hydrophobic domain. The central hydrophobic domain penetrates through the LD matrix. The N and C termini of oleosin polypeptides are located on the surface of the LD in contact with the cytoplasm, and their negatively charged residues provide electrostatic repulsion and steric hindrance, thus keeping the LDs as independent entities [17,20,24]. This structure and topological orientation make LDs and oleosins potentially useful in recombinant proteins [17]. Compared with other expression systems for recombinant proteins using bacteria, fungi, insects, and mammals, a recombinant protein production system using plant/algae has a huge advantage, as they can express heterologous proteins well, effectively, and cheaply, and can produce biologically active proteins [17,111,112,113]. In order to produce these proteins, they need to be targeted to LDs. There are two strategies for carrying out this process. In the first strategy, a recombinant protein can be expressed as a fusion protein to the LDs’ surface oleosin, which will target it to LDs. In the second strategy, the objective protein can be targeted to LDs using a suitable ligand as affinity tags. The abovementioned methods can be used for the production of growth factors, insulin, and vaccines [17,113,114,115].

#### 6.2.1. Growth Factors

Growth factors are active proteins or polypeptide substances that exist in the organism and have a wide regulatory effect on the growth and development of the organism. There are many kinds of growth factors, such as epidermal growth factor (EGF), fibroblast growth factor (FGF), insulin-like growth factor (IGF), interleukin (IL), nerve growth factor (NGF), etc. They can bind with the specific receptor of cell membranes and play an important role in regulating human immunity, hematopoiesis, nerve and hair follicle regeneration, wound healing, blood vessel formation, cell differentiation, and other aspects [111,116,117,118,119,120,121]. Plant LDs, as an expression system, provide a convenient, safe, and effective method for the production of these proteins [111,113,120,121,122,123]. Human insulin-like growth factor 1 (hIGF-1) has been expressed in *Arabidopsis thaliana* seeds via oleosin fusion technology. The expression level of hIGF-1 was about 0.17% of the total seed protein, and the biological activity of the hIGF-1 expressed in plants was confirmed in vitro cell experiments [119]. Recombinant human fibroblast growth factor 9 (rhFGF9) was expressed in the lipid droplets of *Arabidopsis thaliana* and *Carthamus tinctorius* L., and the rhFGF9 produced from plant LDs was confirmed to be able to promote cell proliferation, hair growth, and wound healing [111,113]. Additionally, Guo et al. [122] expressed recombinant acidic fibroblast growth factor (αFGF) in LDs of *Arabidopsis thaliana* and carried out a series of toxicity tests and skin sensitisation test to evaluate the dermal safety of the αFGF produced by the plant expression system. The results suggested that it had no obvious toxicity and skin sensitization in animals.

#### 6.2.2. Insulin

With a rapid increase in diabetic patients globally, the existing techniques for producing insulin using mammalian cells, *Escherichia coli*, and yeast expression systems will not be able to meet the growing demand for affordable insulin due to the limited production capacity and high production cost [124,125,126,127]. Therefore, it is essential to develop new expression systems for producing insulin. Plants have considerable potential for the production of biopharmaceutical proteins and peptides because they are easily converted and provide a cheap source of protein [128,129]. Through the use of LD oleosin fusion technology, the recombinant human precursor of insulin (DesB_30_-insulin) has been expressed in *Arabidopsis thaliana*, and the expression level of the plant-derived protein accounted for 0.13% of the total seed protein [115]. After isolation and purification, recombinant insulin needs to be digested and matured by trypsin before application [130]. Additionally, biological activity tests showed that the plant-derived insulin after enzymatic maturation could lower blood glucose as effectively as Humulin^®®^ and Roche insulin, and there were no toxic side-effects [115].

#### 6.2.3. Vaccines

Artificial lipid nanodroplets (ALNDs) have emerged in the pharmaceutical industry as promising carriers for a variety of therapies [131]. ALNDs can be prepared using a mixture of lipids and proteins through the film hydration, reverse phase evaporation, and microfluidic hydrodynamic focusing techniques [132,133,134]. The ALNDs prepared by surface modification with specific ligands can bind to specific receptors on the cells for more efficient delivery of drugs or vaccines [131,135,136]. Recently, Pfizer/BioNTech and Moderna used the ALNDs as carrier for COVID-19 messenger RNA (mRNA), and successful developed two approved COVID-19 vaccines [137,138,139]. Apart from ALNDs, the LDs extracted from plant seeds also were used to prepare vaccines in the patent of SemBioSys Genetics Inc. (Calgary, AL, Canada) [140]. The patent describes that the antigen can be physically associated with the surface of the LDs in the vaccine’s formulation, or the antigen can be prepared as a recombinant fusion protein with the oleosin which targets the expression of the antigen on the surface of the lipid droplets. Furthermore, the vaccines thus produced can be used to induce an immune response against any antigen by the percutaneous or mucosal immunization routes. The percutaneous immunization approach, in particular, is non-invasive and easy to use, and increases the patients’ compliance, helping to increase vaccination rates and reducing the number of trips to the doctor’s office for vaccinations, thus significantly reducing the healthcare system’s overall costs [140].

#### 6.2.4. Astaxanthin

Astaxanthin is a natural carotenoid with unique antioxidant properties derived from *Haematococcus pluvialis*, yeast, and aquatic organisms [141,142,143]. Astaxanthin is of great research value for the prevention and treatment of oxidative stress-related diseases and has been widely examined [143,144,145]. However, its poor stability and water solubility greatly reduce its bioavailability and limit its practical applications. Therefore, to overcome these limitations, the preparation of ALNDs for use as a carrier has been reported to protect the antioxidant activity of astaxanthin [143,145,146,147,148]. In addition, the LDs isolated from plants and microalgae can be used as a stable oil-core biological carrier. For example, Acevedo et al. [149] used LDs from *Brassica napus* as carriers to stabilize and produce astaxanthin. They found that LD-based astaxanthin was more stable than free astaxanthin. This potential application of LDs will provide a new development direction for drug delivery.

### 6.3. Biofuel Applications

LDs derived from plants and microalgae store neutral lipids as a source of energy and carbon and serve as potential substitutes to fossil fuels. Some plants, such as soybeans, rapeseed, peanut, jatropha and mahua, are already used for the production of biodiesel. However, the low yield and/or the high cost of feedstocks significantly limit its application at a larger scale [150,151]. Moreover, if edible plants are used for biofuels, it would raise concerns about food and feed production systems. A promising alternative strategy is to use non-edible bioenergy crops or vegetative organs as feedstocks for the production of biodiesel after understanding the molecular processes governing oil storage in LDs. The number of LDs and the lipid content in leaves are generally much lower than those in seeds; however, they can be elevated by bioengineering strategies or abiotic stress conditions. For example, through the disruption of *CGI*-58 and *PXA1*, it was possible synthesize more fatty acids and accumulate more TAGs in leaves [4]. Some studies have revealed that the level of TAGs rapidly increased during heat, drought, and salt stress [1,152]. Moreover, compared with traditional food plants, microalgae are regarded as a potential feedstock for biofuel due to their several advantages (high lipid content, rapid growth, and photosynthesis rates, as well as low land usage) [153,154]. Especially under some stresses (nutrient deprivation, temperature, and light), the ability to accumulate TAGs and LDs has been improved for a variety of microalgae [14,153,155]. The content of TAGs could reach up to 37% (*w*/*w*) of dry weight after 11 days of nitrogen starvation in *Nannochloropsis* [156]. Therefore, there is great potential to maximize the lipid content for biofuel applications.

### 6.4. Bioplastic Applications

Although most LDs are rich in TAGs, some other species can accumulate polyhydroxyalkanoates (PHAs) in the LDs [123,157,158]. Similar to the role of TAGs, PHAs are natural polyesters and produced as carbon and energy reserves during the growth process [159,160,161]. PHAs are fully biodegradable, eco-friendly, and renewable plastics that have been developed in recent years [162,163,164]. Compared with petroleum-based polymers, plant/algae-based polymers do not cause lasting environmental harm [158,165], and even show improved mechanical properties [166]. PHA bioplastics are divided into three major groups based on the number of carbon atoms that make up their functional groups: 3–5 carbon atoms (PHA_SCL_), 6–14 carbon atoms (PHA_MCI_), and more than 15 carbon atoms (PHA_LCL_) [167]. The differences result from the substrate specificity of the PHA synthases [157]. Generally, the performance of PHA_LCL_ is close to that of traditional petrochemical-based plastics [168]. Although PHAs occur naturally in microalgae, their percentage by weight is usually relatively low (less than 10% of cellular biomass) compared with other microorganisms such as bacteria [169,170]. Microalgae can accumulate more PHAs under environmental stresses such as N deficiency and P deficiency [171]. It was reported that the accumulation of PHAs in *Scenedesmus* sp. was 8.61% (DW) under normal growth condition, but changes in the nutrient levels (nitrogen, phosphorus, iron, salinity, and glucose) increased it by 3.4-fold [170]. In addition, food waste can be used as a medium for the cultivation of microalgae, which can reduce the production costs of PHAs compared with bacterial fermentation [157,159,172]. The PHAs biosynthesized by microalgae have shown considerable potential for various applications in industries such as packaging polymers in the food industry, bone plates and vascular grafts in the medical industry, and agricultural and horticultural polymers [173,174].

## 7. Limitations of This Review

In this review, we have presented a model for the formation of lipid droplets (LDs) and discussed the current understanding of the molecular mechanisms involved in this process. However, despite significant progress in recent years, our knowledge of LDs’ biology remains incomplete, and there are still many unanswered questions. For instance, the precise mechanisms underlying the localization of the TAG synthesis sites within the ER and how these relate to the formation of LDs are not yet fully understood. Although it has been suggested that TAGs initially form lenses within the ER membrane, this model has not been extensively tested. Moreover, the proteins that participate in the formation and organization of TAG lenses remain largely unknown, and further investigation is needed to clarify these processes.

In addition, recent studies have suggested that LDs play a critical role in cellular processes beyond energy storage, including regulation of the membrane’s lipid composition, modulation of signaling pathways, and sequestration of toxic lipid species. Therefore, a comprehensive understanding of LDs’ biology will require the elucidation of the functional roles of LDs in various cellular contexts, as well as their interactions with other organelles and biomolecules. Furthermore, advances in imaging technologies and proteomic analyses will enable researchers to better visualize and characterize the dynamic nature of LDs and their constituent proteins in living cells.

Overall, although significant progress has been made in understanding LDs’ biology, many questions still remain. Further research is needed to fully elucidate the mechanisms underlying the formation and turnover of LDs, and their functional roles in different contexts.

## 8. Conclusions and Suggestions

LDs are cellular organelles involved in diverse biological processes, and are composed of a neutral lipid core surrounded by a phospholipid monolayer membrane. Measurable progress in the biology of LD has been made by researchers over the past few years. Our understanding of LDs’ biology in plants and microalgae has been enhanced to clarify the formation and turnover of LDs and increase the inventory of LD-associated proteins, as well as to reveal their functions. However, there is a need to address questions for the exploitation of all the potential of LDs. For example, although LDs share common features in organisms, the components and metabolic differences of LDs between different species or strains should also be revealed, and the precise functions and relationships of LDs as well as their associated proteins should be further investigated. Moreover, extracting LDs from plants and microalgae for different applications is also a challenge. Each method has advantages and limitations. It is difficult to indicate which is the best way for all organisms. In general, we need to choose non-toxic methods for food applications of LDs, non-contaminated methods for biological applications of LDs, and low-cost and efficient methods for biofuel applications.

## Figures and Tables

**Table 2:** A summary of the information on lipid droplets from plants and microalgae.

Organisms		LD Size (μm)	Zeta Potential (mV)	Isoelectric Point	References
Plants	Soybean	0.2–0.5	−20 to +12	Around pH 4	[28,54]
	Peanuts	0.6–5.4	−18 to −8	Around pH 5	[55]
	Sunflower seed	0.3–13	−22 to −9	pH 5–6	[30,55]
	Maize	0.95–2.55	−16.4 to 23.3	pH 4.6–4.8	[56]
	*Paeonia ostia seed*	0.4–1.2	−50 to −35	Na*	[57]
	Rapeseed	0.2–6	−65 to +55	pH 5–7	[58]
	Coconut	1–20	−33.8 to −13.0	<pH 6.1	[31]
Microalgae	*Eremosphaera viridis*	1–2.5	Na*	Na*	[59]
	*Chlorella sp.*	0.1–5.0	Na*	Na*	[60]
	*Chlamydomonas reinhardtii*	1.7–2.5	Na*	Na*	[61]
	*Dunaliella salina*	0.5–0.8	Na*	Na*	[33]
	*Phaeodactylum tricornutum*	1–2.5	Na*	Na*	[62]
	*Thraustochytrid*	0.1–1	−57 to +4	Around pH 3	[14]

Na*: not available.

**Table 4:** The advantages and limitations of different physical methods.

Physical Methods	Advantages	Limitations
Grinding	Easy to operate	Low rupture efficiency; time-consuming
Pressing	Easy to set up and operate; applicable on a large scale	Low rupture efficiency
High-pressure homogenization	Low operational cost; possibility of upscaling; low risk of thermal degradation	High energy consumption
Ultrasound	Reduced extraction time	High energy consumption; lead to denaturing of the intracellular components; scaling up is not suitable
Microwave	Reduced extraction time; high efficiency with superior quality; possibility of upscaling	High energy costs of scaling up
Bead beating	Easy to operate; simplicity of the equipment	Low rupture efficiency; time-consuming; not suitable for scaling up is not suitable
Pulsed electric field	Low energy consumption; fast and efficient Possibility of up-scaling	Sensitive to the conductivity of the medium

## Data Availability

Not applicable.
